# Properties, Production, and Recycling of Regenerated Cellulose Fibers: Special Medical Applications

**DOI:** 10.3390/jfb15110348

**Published:** 2024-11-16

**Authors:** Sandra Varnaitė-Žuravliova, Julija Baltušnikaitė-Guzaitienė

**Affiliations:** Department of Textile Technologies, Center for Physical Sciences and Technology, Demokratų Str. 53, LT-48485 Kaunas, Lithuania; julija.baltusnikaite@ftmc.lt

**Keywords:** regenerated cellulose fibers, medical, viscose, lyocell, modal, recycling

## Abstract

Regenerated cellulose fibers are a highly adaptable biomaterial with numerous medical applications owing to their inherent biocompatibility, biodegradability, and robust mechanical properties. In the domain of wound care, regenerated cellulose fibers facilitate a moist environment conducive to healing, minimize infection risk, and adapt to wound topographies, making it ideal for different types of dressings. In tissue engineering, cellulose scaffolds provide a matrix for cell attachment and proliferation, supporting the development of artificial skin, cartilage, and other tissues. Furthermore, regenerated cellulose fibers, used as absorbable sutures, degrade within the body, eliminating the need for removal and proving advantageous for internal suturing. The medical textile industry relies heavily on regenerated cellulose fibers because of their unique properties that make them suitable for various applications, including wound care, surgical garments, and diagnostic materials. Regenerated cellulose fibers are produced by dissolving cellulose from natural sources and reconstituting it into fiber form, which can be customized for specific medical uses. This paper will explore the various types, properties, and applications of regenerated cellulose fibers in medical contexts, alongside an examination of its manufacturing processes and technologies, as well as associated challenges.

## 1. Introduction

Civilizations dating as early as the ancient Egyptians used natural materials such as flax, silk, hemp, and cotton to create sutures and bandages for wound healing. Some natural fibers are still in use today because of their intricate and versatile structures, favorable biocompatibility, and abundance.

In recent years, the definition of “textile” has broadened, necessitating an emphasis on the increasing significance of diverse textile materials for applications in technology, industry, construction, architecture, agriculture, and medicine [1,2,3]. The medical textile segment is regarded as the most rapidly expanding sector within the technical textile industry [4,5,6]. Textile materials, in conjunction with scientific methodologies, are extensively utilized in medical and surgical applications due to their durability, versatility, convenience, and antimicrobial properties [7]. These polymers should be dissolvable or meltable for extrusion, and their molecular chains should be linear, long, flexible, and capable of orientation and crystallization.

Medical textiles are engineered for various medical uses, including both internal and external applications, with the capability of being implantable or non-implantable. They are utilized in biological structures for the assessment, treatment, enhancement, or regeneration of tissues, organs, or physiological functions. Examples include plasters, dressings, bandages, and compression garments [8].

Ensuring a sustainable world from the textile perspective has motivated the usage of sustainable and biodegradable materials as alternatives.

### 1.1. Textile Fibers for Medical Applications

Polysaccharide-based fibers, particularly cellulose, are widely used in medical applications such as wound dressings and hemostats due to their thrombogenic properties [9]. As a natural biopolymer, cellulose is an integral component of plant cell walls and is synthesized by specific microbes, making it the most abundant organic substance on Earth. This abundance, coupled with properties like biocompatibility, biodegradability, and renewability, makes cellulose an attractive choice for designing biomaterials in biomedical fields.

Cellulose, derived from various natural sources, such as cotton and woody biomass, varies in content; cotton has up to 90% cellulose, making it nearly pure, while woody biomass contains about 40% to 50% cellulose [10]. Its structural strength comes from β-1,4-glycosidic linkages that form long chains of D-glucose units. These properties are harnessed in the creating of advanced healthcare products like aerogels, hydrogels, films, and fillers [11,12].

Incorporating cellulose into medicated textiles and pharmaceutical products fosters the development of new healthcare approaches, aligning with the goal of reducing the medical sector’s impact by substituting non-biodegradable waste with sustainable, green polymers. This aligns with the wider movement towards sustainability in material science, emphasizing the critical role of naturally occurring substances in modern applications.

The regeneration process transforms the cellulose chain conformation from cellulose I to cellulose II, resulting in a structure with more amorphous regions and enhanced crystallinity [13]. This change also facilitates extensive modifications in regenerated cellulose (RC) products, including hydrogels, aerogels, cryogels, xerogels, membranes, thin films, and fibers [14]. The most commonly used structures in medical textiles are fibers [15,16,17]. Fibers are the primary materials for the textile industry, which vary in type based on their origin and chemical composition, as natural and chemical (man-made and synthetic). Regenerated cellulose fibers are paramount in the medical textile industry due to their exceptional biocompatibility, biodegradability, and mechanical properties. These fibers, derived from natural cellulose, undergo various chemical processes to transform their structure, making them suitable for diverse medical applications.

Cellulose can be regenerated to produce films/membranes, hydrogels/aerogels, filaments/fibers, microspheres/beads, bioplastics, etc., which show potential applications in textiles, biomedicine, energy storage, packaging, etc. Importantly, these cellulose-based materials can be biodegraded in soil and oceans, reducing environmental pollution [18]. The cellulose solvents, dissolving mechanism, and strategies for constructing the regenerated cellulose functional materials with high strength and performances, together with the current achievements and urgent challenges, are summarized, and some perspectives are also proposed.

There are five conventional types of regenerated cellulose or cellulose-derived fibers: viscose; lyocell; cupro; acetate; and modal. These fibers have been widely recognized for their biocompatibility, moisture retention capabilities, and biodegradability, making them suitable candidates for various medical applications, including wound care and tissue scaffolding. Recent innovations have focused on improving their structural properties, antimicrobial functionality, and integration with bioactive agents. These developments have opened new avenues for more efficient and sustainable healthcare solutions. The advantages of regenerated cellulose fibers in medical applications compared to synthetic fibers (like polypropylene and polyester) and natural fibers (like cotton or silk) are presented in Table 1. 

**Figure 1:**
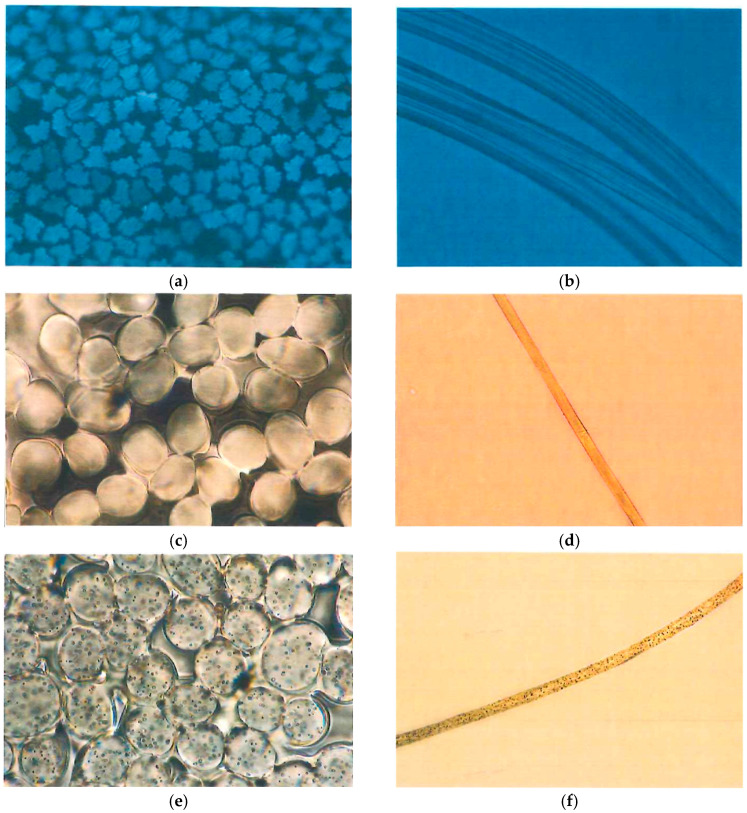

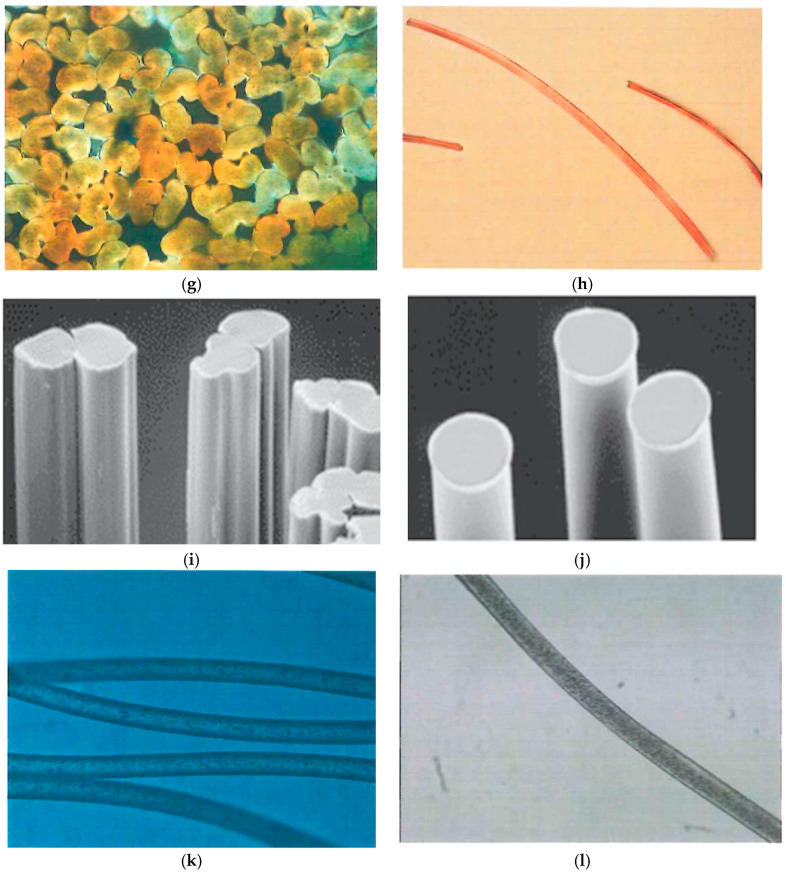
The microscopic views of regenerated cellulose fibers and some common synthetic fibers [24,25]: (**a**) microscopic view of viscose fiber cross-section; (**b**) microscopic longitudinal view of viscose fiber; (**c**) microscopic view of lyocell fiber cross-section; (**d**) microscopic longitudinal view of lyocell fiber; (**e**) microscopic view of cupro fiber cross-section; (**f**) microscopic longitudinal view of cupro fiber; (**g**) microscopic view of modal fiber cross-section; (**h**) microscopic longitudinal view of modal fiber; (**i**) microscopic view of acetate fiber; (**j**) microscopic view of typical melt spun synthetic fibers cross-section, i.e., polyester, polyamide, and olefin; (**k**) microscopic longitudinal view of polyester fiber; (**l**) microscopic longitudinal view of polyamide fiber; ((**a**–**h**,**k**,**l**) Reprinted with permission from Ref. [24]. Copyright 2012 Lithuanian Standards Board). ((**i**,**j**) Reprinted with permission from Ref. [25]. Copyright 2008 Elsevier).

The microscopic views of regenerated cellulose fibers and some common synthetic fibers are presented in Figure 1. More microscopic views of different fibers may be found in [24].

### 1.2. The Classification of These Different Regenerated Cellulose Fibers Is Based on Fiber Production Method

- Viscose fibers

Viscose fibers are derived from natural cellulose, primarily sourced from wood pulp or cotton linters. The process of converting cellulose into viscose involves several chemical treatments, including steeping, shredding, and xanthation, resulting in fibers that closely resemble natural fibers like cotton and silk in texture and appearance. Viscose is appreciated for its versatility and biodegradability, making it a popular choice in various textile applications [26]. The development of viscose fibers dates back to the late 19th century when researchers sought to create a fiber that mimics the properties of natural silk. The viscose process was first patented by British chemists Charles Cross, Edward Bevan, and Clayton Beadle in 1892, marking the beginning of commercial production in the early 20th century [27].

Viscose rayon, the oldest and most widely used regenerated cellulose fiber, is produced by dissolving cellulose in a solution of sodium hydroxide and carbon disulfide to form cellulose xanthate. This solution is then extruded into fibers and regenerated in an acidic bath [28,29]. Viscose rayon is highly absorbent and soft, making it ideal for applications such as wound dressings, bandages, and surgical swabs. Its excellent sterilizability further enhances its suitability for medical applications [30,31].

Viscose rayon fibers typically have a round or slightly irregular cross-sectional shape. The cross-sectional geometry of viscose fibers is largely influenced by the coagulation conditions during the spinning process, which can lead to variations in shape and size. Studies have shown that these fibers often exhibit a circular or slightly oval cross-section depending on the specific conditions under which they are formed [32,33]. The internal structure often shows a central lumen (a hollow core) surrounded by a cellulosic wall with varying degrees of fibrillation. This lumen, a characteristic feature of regenerated cellulose fibers like viscose, is formed due to the rapid coagulation of the cellulose solution during spinning, which traps air or other gasses within the fiber structure [34]. The cellulosic wall surrounding the lumen is composed of aligned cellulose microfibrils, which can vary in their degree of crystallinity and orientation, affecting the fiber’s mechanical properties [35]. The cross-section of viscose rayon appears with a smooth, even texture but can exhibit some degree of irregularity due to the manufacturing process. Variations in coagulation conditions, such as temperature and solvent concentration, can lead to irregularities in the fiber cross-section, contributing to a less uniform appearance [36,37]. This irregularity can also be attributed to fluctuations in the flow rate during extrusion, which affects the fiber’s final shape. In the longitudinal view, viscose rayon fibers display a smooth surface with longitudinal striations or fibrils along their length. These striations result from the stretching and drawing processes during fiber production, which align the cellulose chains and create a characteristic fibrillar texture [32]. The surface of viscose fibers may also exhibit undulations or waviness, which are linked to the mechanical drawing and relaxation steps that the fibers undergo during spinning. The fibers are generally smooth but can show some periodic variations in diameter due to variations in spinning conditions. These variations in diameter are primarily caused by inconsistencies in the extrusion and coagulation phases, where factors like pressure, temperature, and draw ratio play significant roles [33]. The periodic changes in diameter can impact the fiber’s overall mechanical performance and its suitability for various applications.

Recent advancements in viscose rayon technology have focused on improving fiber strength and reducing environmental impacts. For instance, innovations in the viscose process have led to the development of more sustainable production methods and enhanced fiber properties [38].

For medical textiles, viscose fibers may be engineered to have specific structural properties, such as enhanced porosity, higher purity, and tailored surface characteristics, to improve their performance in medical applications. Medical-grade viscose fibers require higher purity levels to avoid any adverse reactions. This involves more rigorous washing and bleaching processes to remove any residual chemicals or impurities [33]. The fiber structure can be adjusted to enhance porosity and surface area, which is critical for applications like wound dressings, where absorbency and moisture management are crucial. Viscose fibers for medical use may be coated with antimicrobial agents or other functional substances to provide additional benefits such as infection control [39,40].

- Lyocell

Lyocell fibers are a type of regenerated cellulose fiber known for their eco-friendly production process and superior performance characteristics. Lyocell is a form of rayon, primarily derived from wood pulp. Unlike other types of rayon, such as viscose, the production of lyocell involves an environmentally friendly process that uses a non-toxic solvent, N-methylmorpholine N-oxide (NMMO). Introduced in the 1990s, lyocell has quickly gained popularity due to its sustainability and high performance [41,42]. The development of lyocell fibers was driven by the need for a more sustainable alternative to traditional rayon fibers. The first commercial production of lyocell was by Courtaulds Fibers under the brand name Tencel in the early 1990s [33].

Lyocell fibers are renowned for their distinctive structural and mechanical properties, which set them apart from other regenerated cellulose fibers such as viscose. The cross-sectional profile of lyocell fibers is typically round and exhibits a smooth, uniform surface. Unlike viscose fibers, which often display a central lumen, lyocell fibers generally have a more compact and dense cellulosic structure with minimal or no lumen. This compact structure results from the solvent-spinning process used in lyocell production, where cellulose is dissolved in N-methylmorpholine N-oxide (NMMO) and then extruded and regenerated in a controlled manner [41,43]. The uniform and round cross-section of lyocell fibers contribute to their high tensile strength and smooth texture, making them particularly well suited for applications requiring durability and a fine hand feel. The absence of significant internal voids, such as a pronounced lumen, and the alignment of cellulose microfibrils during the spinning process result in fibers that are not only strong but also resistant to deformation. This is a crucial advantage in textile applications, where fiber consistency and performance are critical [33]. In terms of surface characteristics, lyocell fibers are characterized by their smooth and even surface, which is evident in both cross-sectional and longitudinal views. The spinning process ensures that the fibers have fewer surface defects compared to viscose fibers, which often show more pronounced striations and irregularities. The smooth surface of lyocell fibers contributes to their luxurious feel and low surface friction, making them ideal for high-end textiles, including apparel and home textiles. Moreover, this smoothness enhances the fibers’ dyeing properties, allowing for vibrant and uniform coloration [44]. The longitudinal view of lyocell fibers further emphasizes their superior quality. The fibers are generally straight with a high degree of smoothness and uniformity. This straightness, combined with the fiber’s inherent strength, reduces the likelihood of pilling and wear, which is a common issue with less uniform fibers. The enhanced mechanical properties of lyocell fibers, including their high tensile strength and durability, make them suitable for a wide range of applications, from fashion textiles to technical fabrics [45].

Lyocell fibers are produced using the N-methylmorpholine N-oxide (NMMO) process, which is considered more environmentally friendly compared to the viscose process [42,46]. The NMMO solvent effectively dissolves cellulose, which is then extruded into fibers and regenerated. Lyocell fibers exhibit high tensile strength, excellent moisture management, and biocompatibility, making them suitable for surgical gowns, drapes, and wound dressings [26,47].

Similarly to viscose, lyocell fibers intended for medical textiles undergo specific modifications and enhanced production methods to meet medical standards. Lyocell fibers for medical use are often engineered to have properties such as higher absorbency, enhanced biocompatibility, and tailored mechanical strength. Ensuring biocompatibility involves minimizing any potential cytotoxicity. This can be achieved by optimizing the fiber’s chemical composition and ensuring the absence of harmful residual solvents [33]. Medical applications may require fibers with higher tensile strength and durability, especially for sutures or implants [48].

Recent studies have highlighted the advantages of lyocell fibers in medical textiles due to their enhanced mechanical properties and environmental sustainability. The lyocell process continues to evolve, with research focusing on improving fiber functionality and reducing environmental impacts [42]. The ongoing evolution of the lyocell process has focused on improving fiber functionality, such as controlling fibrillation and enhancing the durability of the fibers for demanding medical applications like wound dressings and surgical textiles. Advances in the lyocell process include the use of cross-linking agents and enzymatic treatments to minimize fibrillation, thus improving fiber performance in medical applications [49,50].

Moreover, the lyocell process continues to innovate with research aimed at further reducing its environmental impact, including the development of more efficient solvent recovery systems and the exploration of alternative raw materials for cellulose production [51,52].

- Modal

Modal fibers are a subtype of rayon specifically designed to improve upon the properties of regular viscose rayon. They are made from beech tree pulp and undergo a series of chemical processes to achieve their unique qualities. Modal fibers are often praised for their luxurious feel and durability, making them a popular choice in the textile industry.

Modal fibers were developed as a response to the need for a more robust and high-performing type of rayon. The name “modal” was coined by the Austrian company Lenzing AG, which remains one of the leading producers of these fibers [53,54]

Modal fibers, a type of regenerated cellulose fiber, are produced through a process similar to viscose but with modifications that enhance fiber strength and elasticity [52]. Modal fibers are known for their softness, durability, and resistance to shrinkage. These properties make them suitable for reusable medical textiles, such as hospital bed linens and patient clothing [47].

Modal fibers often have a slightly oval cross-section. The internal structure is similar to viscose but with improved structural integrity and uniformity due to modifications in the production process. The cross-sectional view shows a more consistent shape and density compared to standard viscose fibers. Modal fibers exhibit a smoother and more consistent surface compared to viscose. The longitudinal view shows a straight and uniform appearance with fewer surface irregularities. The fibers are typically smooth, with fewer longitudinal striations compared to viscose fibers. [55]

Modal fibers are increasingly being used in combination with other fibers to create high-performance textiles for medical applications. Research has shown that modal fibers can be blended with antimicrobial agents to enhance their functionality in medical textiles [56].

- Cupro (Cuprammonium Rayon)

Cupro fibers are derived from cellulose, specifically cotton linter, which is a byproduct of the cotton industry. The production of cupro fibers involves dissolving cellulose in a copper–ammonium solution, hence the name cuprammonium rayon [26,57].

Cupro fibers are produced using the cuprammonium process, where cellulose is dissolved in a copper–ammonium solution [58]. These fibers are smooth, lustrous, and hypoallergenic, making them suitable for applications requiring non-abrasive materials, such as advanced wound care products [59,60]

Recent advancements in the cuprammonium process have focused on improving fiber strength and exploring new applications in medical textiles. Cupro fibers are being studied for their potential use in high-performance medical textiles due to their unique properties.

Cupro fibers typically have a round or slightly irregular cross-sectional shape. The internal structure is dense and smooth, with minimal lumen. The fibers have a smooth and even cross-section, reflecting their fine, high-quality texture. In the longitudinal view, cupro fibers display a very smooth surface with minimal striations or surface defects. The fibers are straight and exhibit a high level of smoothness and sheen [60,61].

Cupro (cuprammonium rayon) fibers are characterized by their silk-like feel and high moisture absorption. For medical uses, the fibers are often engineered to enhance their biocompatibility and reduce potential allergic reactions.

- Acetate

Acetate fibers, also known as cellulose acetate fibers, are derived from cellulose. They are produced by acetylating the cellulose extracted from wood pulp or cotton linters. Acetate fibers are unique due to their blend of natural and synthetic properties, which provide distinct advantages in textile manufacturing [26,62].

Acetate fibers are produced through the acetylation of cellulose, where cellulose is reacted with acetic anhydride or acetyl chloride in the presence of a catalyst. This process yields a fiber with distinct properties compared to other regenerated cellulose fibers. Acetate fibers are characterized by their smooth texture, lustrous appearance, and relatively low moisture absorption [63].

Acetate fibers are generally biocompatible, which makes them suitable for certain medical applications. Their smooth surface can reduce irritation in contact with the skin. In addition, acetate fibers are known for their soft and silky feel, which can enhance comfort in medical textiles like patient clothing and surgical drapes. This type of fiber can be sterilized, making them suitable for medical textiles that require sterilization before use [64,65,66].

While acetate fibers are not as commonly used as some other regenerated cellulose fibers in medical textiles, they possess properties that make them suitable for specific applications. Their softness, biocompatibility, and potential for customization through chemical treatments can make them a viable option for certain medical textile products. Regenerated cellulose fibers (RCFs) offer a solution to the drawbacks of synthetic fibers while preserving the benefits of natural fibers. According to The Materials Market Report 2023 by Textile Exchange [67], the RCFs covered 6% (i.e., 7,3 mln. tons of fibers) of a global market in 2022; among that, viscose fiber production accounted for 80% of all the RCFs and a production volume of around 5.8 million tons in 2022. Acetate covered 13% of all the RCF market, and lyocell, modal, and cupro, respectively, 4%, 3%, and 0.2%. Along with that, 0.5% of all RCF market shares recycled regenerated cellulose fiber producers.

## 2. Production of RC

RCFs have attracted attention because they can be easily processed via fiber spinning to produce man-made fibers using bio-based feedstocks rather than the cases of natural fibers [68]. There are several methods for producing high-quality regenerated cellulose (RC) fibers through dissolution and regeneration processes [69,70]. Typically, these fibers are created using the spinning method. One of the most common techniques is wet spinning, where a cellulose solution is extruded through a spinneret into an acid bath for coagulation. Following this, the fibers are washed first with hot water and then with distilled water to remove salts formed during coagulation. The resulting RC fibers are then dried at room temperature [71].

The properties of regenerated cellulose (RC) fibers can be influenced by different solvent systems [68]. Each solvent system impacts the final characteristics of the RC fibers and offers distinct advantages. The solubility and spinnability of cellulose are influenced by multiple factors, including solvent properties (solvating power and viscosity), raw material characteristics (degree of polymerization, molecular structure, and chemical composition), process parameters (such as temperature, duration, and pressure), and the equipment used for dissolution and spinning [72,73].

The structural and mechanical properties of RC fibers can be optimized by adjusting parameters, such as draw ratio, spinning dopes, and the spinning process. Research [71,74] shows that at low draw ratios, regenerated cellulose (RC) fibers display an irregular morphology with pronounced surface grooves. Conversely, when fibers are produced at high draw ratios, they have a smoother surface and a more rounded structure.

High-purity cellulose pulp is essential for producing medical-grade viscose fibers. Advanced purification techniques, such as enzymatic treatment and bleaching, are employed to remove impurities and ensure biocompatibility [6,75,76].

### 2.1. Production Methods of Viscose Fibers

The production of viscose fibers involves several stages, including the preparation of cellulose pulp, dissolution, regeneration, and finishing. Specific production methods tailored for medical textiles focus on enhancing the purity and functionality of the fibers.

The first step in producing viscose fibers is extracting cellulose from wood pulp or cotton linters. The pulp undergoes purification to remove hemicellulose, lignin, and other impurities [77,78,79]. The purified cellulose is then treated with sodium hydroxide (NaOH) to form alkali cellulose. This process increases the cellulose’s reactivity, preparing it for the subsequent chemical reactions [80]. The alkali cellulose is aged under controlled conditions, typically for 1–2 days. Aging reduces the degree of polymerization, which is necessary for achieving the desired viscosity in the final viscose solution [81]. Aging is followed by the xanthation process, where the alkali cellulose reacts with carbon disulfide (CS2) to form cellulose xanthate. This reaction imparts solubility to the cellulose in aqueous sodium hydroxide. The cellulose xanthate is dissolved in dilute sodium hydroxide to produce a viscous orange-yellow solution known as viscose. The solution is allowed to ripen for a specific period, enabling the cellulose chains to rearrange and achieve the required spinnability [29]. The ripened viscose solution is filtered to remove undissolved particles and degassed to eliminate air bubbles. These steps are crucial for ensuring the uniformity and quality of the fibers [82]. 

The viscose solution is extruded through a spinneret into an acid bath (usually sulfuric acid), where the cellulose is regenerated into solid fibers through a process of acid precipitation and coagulation. This step determines the final properties of the viscose fibers [83,84]. Viscose rayon fibers can be produced in two forms: continuous filament and staple fibers, but generally, viscose rayon is manufactured as staple fiber.

After regeneration, the fibers undergo various post-treatment processes, including washing, bleaching, and finishing. These treatments enhance the fibers’ properties, such as whiteness, strength, and softness [70,85].

The production of viscose fibers involves the use of chemicals such as sodium hydroxide and carbon disulfide, which can pose environmental and health risks. However, advancements in production technologies aim to mitigate these impacts through improved chemical recovery and waste management systems [84,86].

The production methods of viscose fibers for medical textiles often incorporate additional steps to ensure the fibers meet medical standards. Medical-grade viscose fibers must be sterilizable. The production methods may include steps that ensure fibers can withstand sterilization processes, such as autoclaving, gamma irradiation, or ethylene oxide treatment. The entire production process is subjected to more rigorous quality control to ensure the fibers are free from contaminants and have consistent properties suitable for medical use [87,88].

**Figure 2:**
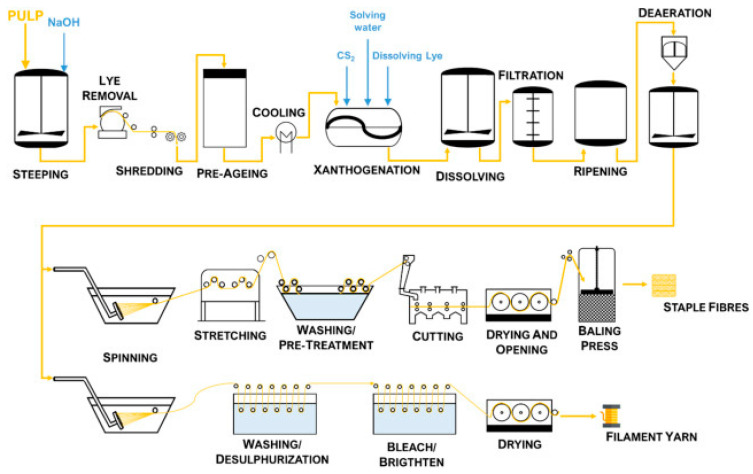
Production scheme of viscose fibers [89] (Reprinted with permission from Ref. [89]. Copyright 2021 Elsevier).

The principal production scheme of viscose fibers is presented in Figure 2.

### 2.2. Production Methods of Lyocell Fibers

The production of lyocell fibers involves several key stages: cellulose extraction, dissolution, spinning, and post-treatment. The most distinguishing feature of lyocell production is the use of N-Methylmorpholine N-oxide (NMMO) as the solvent, which is non-toxic and recyclable.

The primary raw material for lyocell production is wood pulp from sustainably managed forests. The pulp is purified to remove lignin and hemicellulose, resulting in high-purity cellulose suitable for dissolution [90]. In the lyocell process, purified cellulose is dissolved directly in an aqueous solution of NMMO. This solvent system is highly effective in dissolving cellulose with no chemical derivatization, distinguishing lyocell from other types of rayon [46,91,92].

The cellulose-NMMO solution, known as the spinning dope, is filtered to remove undissolved particles and air bubbles. The filtered solution is then extruded through a spinneret into a coagulation bath, typically water or a dilute NMMO solution, where the cellulose fibers are regenerated [33,93]. After regeneration, the lyocell fibers undergo several post-treatment steps, including washing to remove residual NMMO, drying, and finishing. These steps are crucial for enhancing the mechanical properties and appearance of the fibers [91]. A significant advantage of the lyocell process is the efficient recovery and reuse of NMMO. Over 99% of the solvent is typically recovered and recycled, minimizing environmental impact and production costs [43,94].

The production of lyocell fibers is considered more environmentally friendly compared to other types of rayon due to the closed-loop solvent recovery system and the use of non-toxic chemicals. Additionally, lyocell fibers are biodegradable and derived from renewable resources, aligning with principles of sustainable development [15,70,95].

The production process for medical-grade lyocell fibers often includes additional purification and validation steps to meet stringent medical standards. The NMMO solvent recovery process is optimized to ensure that no toxic residues are present in the fibers. This is critical for medical applications where patient safety is paramount. Additional washing and bleaching steps are implemented to achieve the high purity required for medical use [96,97].

**Figure 3:**
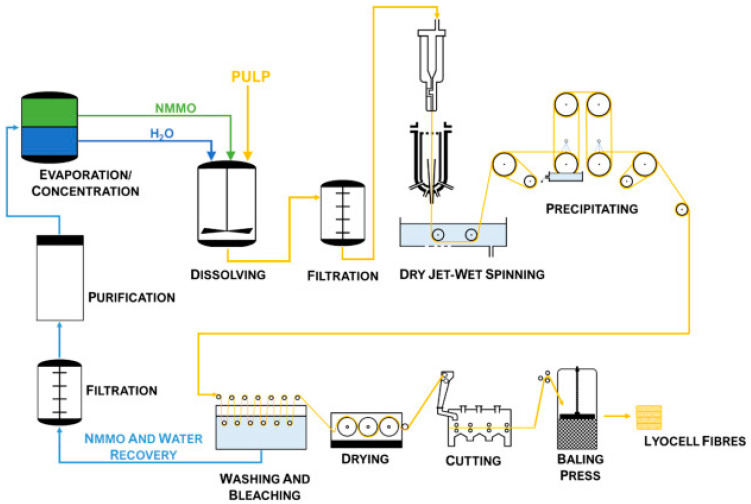
Production scheme of lyocell fibers [89] (reproduced with permission from Elsevier, *Carbohydrate Polymers*; published by Elsevier, 2021).

The principal production scheme of lyocell fibers is presented in Figure 3.

### 2.3. Production Methods of Modal Fibers

The primary raw material for modal fibers is beech wood pulp, which is processed to remove lignin and hemicellulose, resulting in high-purity cellulose [52,98].

The purified cellulose is treated with sodium hydroxide to form alkali cellulose, which is then reacted with carbon disulfide to form cellulose xanthate. This process is similar to the viscose process but is optimized for better fiber strength and quality [99,100,101].

The cellulose xanthate is dissolved in a dilute sodium hydroxide solution to form a viscous solution. This solution is further processed to ensure uniformity and the desired viscosity for spinning.

The spinning dope is filtered and degassed before being extruded through a spinneret into a coagulation bath, typically containing sulfuric acid. The coagulation process regenerates the cellulose, forming solid fibers [33,102].

After spinning, the fibers undergo several post-treatment processes, including washing to remove residual chemicals, bleaching to achieve desired whiteness, and finishing treatments to enhance fiber properties [50].

The production of modal fibers is considered more environmentally friendly compared to traditional viscose rayon. This is primarily due to the closed-loop process used by manufacturers like Lenzing, which recycles chemicals and reduces waste.

Beech wood, the primary source of cellulose for modal fibers, is typically sourced from sustainably managed forests, ensuring a renewable supply of raw materials [103,104].

The production process includes the efficient recovery and recycling of chemicals, such as sodium hydroxide and carbon disulfide, minimizing environmental impact [91].

The production of modal fibers for medical applications may involve additional purification steps to remove any residual chemicals and impurities that could cause adverse reactions. This can include more rigorous washing and bleaching processes. Special finishes or coatings may also be applied to improve antimicrobial properties [105].

### 2.4. Production Methods of Cupro Fibers

The primary raw material for cupro fibers is cotton linter, which is a short fiber left on the cottonseed after the longer cotton fibers have been removed. These linters are purified to remove impurities, resulting in high-purity cellulose [77].

The purified cellulose is dissolved in a cuprammonium solution, which is a mixture of copper(II) hydroxide and aqueous ammonia. This process creates a viscous solution that can be extruded to form fibers. The dissolution process is unique to cupro fibers and differs significantly from other cellulose regeneration methods, like the viscose process [55,106].

The cellulose solution is extruded through a spinneret into a coagulation bath, where the cellulose is regenerated, forming solid fibers. The bath typically contains a dilute sulfuric acid solution, which precipitates the cellulose from the solution.

Cupro fibers undergo post-treatment processes that involve washing to eliminate residual chemicals, bleaching for the desired whiteness, and further treatments to improve fiber properties. The fibers are also stretched to align the cellulose molecules, which improves their strength and uniformity.

The production of cupro fibers involves the use of copper and ammonia, which require careful handling and disposal to minimize environmental impact. Modern cupro production processes include systems for recovering and reusing these chemicals to improve sustainability [107].

To ensure safety and sterility, the manufacturing process for medical-grade cupro fibers includes additional steps to eliminate any residual copper ions and other potentially harmful substances. Enhanced purification and finishing processes are employed to achieve the required medical standards [108].

### 2.5. Production Methods of Acetate Fibers

The main raw materials for acetate fibers are wood pulp and cotton linters. These materials are purified to remove lignin, hemicellulose, and other impurities, yielding high-purity cellulose [109].

The purified cellulose is then subjected to acetylation, where it is reacted with acetic acid and acetic anhydride in the presence of a catalyst (usually sulfuric acid). This reaction converts the hydroxyl groups in the cellulose into acetyl groups, forming cellulose acetate [110].

The cellulose acetate is then dissolved in a suitable solvent, typically acetone, to form a viscous solution. This solution is prepared for the spinning process, where it will be extruded to form fibers [111].

The spinning dope (cellulose acetate solution) is extruded through a spinneret into a coagulation bath or directly into the air (dry spinning). In the coagulation bath, the solvent is removed, and the cellulose acetate fibers solidify. In dry spinning, the solvent evaporates as the fibers solidify [112].

After spinning, the fibers undergo post-treatment processes such as washing to remove residual solvents, stretching to orient the polymer chains, and finishing treatments to enhance properties like dyeability and luster. The production of acetate fibers involves the use of solvents and chemicals that require careful handling and disposal to minimize environmental impact. Advances in solvent recovery and recycling technologies have helped to improve the sustainability of acetate fiber production. Sustainability efforts include sourcing cellulose from sustainably managed forests and using eco-friendly solvents and catalysts during the acetylation and dissolution processes [6].

Modern production processes include systems for recovering and reusing solvents like acetone, which reduces waste and environmental impact. Additionally, the development of biodegradable acetate fibers contributes to their environmental sustainability [113,114].

The production of acetate fibers for medical textiles includes a rigorous purification process to remove any acetic acid and other residual chemicals. Additional treatments may be applied to impart antimicrobial properties, which are crucial for preventing infections in medical settings. This often involves coating the fibers with antimicrobial agents [9].

In recent years, researchers have investigated alternative solvents for cellulose dissolution, with substantial research and development concentrated on a category of solvents known as ionic liquids (ILs). These solvents are considered environmentally sustainable and have demonstrated suitability for spinning regenerated cellulose fibers [115].

Ionic liquids (ILs) are salts that melt below 100 °C and exhibit unique properties such as low vapor pressure, high thermal stability, and a strong ability to dissolve various organic and inorganic substances. These liquids dissolve cellulose by disrupting its hydrogen bonding network, a process that does not require derivatization. Commonly, the ILs used for this purpose feature cations like imidazolium, pyridinium, or ammonium, which are paired with anions such as chloride or acetate. Once dissolved, the cellulose can be regenerated into fibers through a coagulation bath. Often regarded as green solvents, ILs are non-volatile and recyclable, although their cost and biodegradability can vary widely [116].

Another promising process for cellulose dissolution is the urea/alkali process. The urea/alkali process, utilizing a mixture of urea and sodium hydroxide, offers a promising method for cellulose dissolution. In this process, urea serves as a hydrogen bond acceptor, effectively disrupting the hydrogen bonds within cellulose to facilitate dissolution, typically at temperatures below 0 °C, which enhances energy efficiency. This method not only stabilizes the cellulose solution but also is environmentally friendly due to the non-toxic nature of the solvents. However, to achieve commercial viability, this process still requires the optimization of conditions and parameters to enhance the mechanical properties of the fibers. Compared to ionic liquids, scaling this process for large-scale industrial applications presents challenges in terms of efficiency and scalability.

The CarbaCell process is a cellulose dissolution method that uses organic carbonates, such as dimethyl carbonate (DMC), as the primary solvent. This process is noted for its low toxicity and environmental friendliness, as DMC is a biodegradable and less hazardous solvent compared to traditional solvents like carbon disulfide (CS2) used in the viscose process. The CarbaCell process dissolves cellulose by activating the hydroxyl groups of the cellulose molecules with organic carbonates, which facilitate the breakdown of intermolecular hydrogen bonds within cellulose. The resulting cellulose solution can be regenerated into fibers through processes such as wet or dry-jet wet spinning [55].

Each of these processes offers distinct advantages for cellulose dissolution and regeneration into fibers. The CarbaCell process, in particular, stands out due to its low environmental impact and mild operating conditions, utilizing low-toxicity organic carbonates that are both biodegradable and easily recoverable. This makes it an attractive option for sustainable fiber production [117]. Ionic liquids (ILs), on the other hand, are highly effective solvents for cellulose dissolution, but they can be costly and have varying environmental profiles depending on the specific IL used. While ILs provide efficient dissolution and can be recycled, their biodegradability and overall environmental impact need careful consideration [118]. The urea/alkali process is also a promising method because of its low toxicity and cost-effectiveness, making it a sustainable option for cellulose processing. However, it may face challenges in terms of scalability and efficiency compared to ILs and CarbaCell [119].

## 3. Properties of Main Types Regenerated Cellulose Fibers

Cellulose fibers stand out among all the textile fibers for their vast diversity in structure and properties. Natural cellulose fibers feature highly crystalline fibrillar structures arranged in various helical patterns, while fibrils in regenerated cellulose are less in ordered arrangement, and exhibit a wide range of structural variations. Chemically altered and regenerated through different processes, regenerated cellulose fibers, such as viscose rayon, modal, lyocell (Tencel), acetate, and cupro (cuprammonium), are widely used in the textile industry and for medical applications due to their unique properties. The structural diversity of regenerated cellulose fibers results in a broad spectrum of properties (see Table 2) and applications [54].

Regenerated cellulose fibers are composed of cellulose—a natural polymer of glucose. The chemical properties of regenerated cellulose fibers are influenced by the degree of substitution and the method of regeneration, e.g., viscose rayon fibers often feature a lower degree of polymerization and higher susceptibility to degradation compared to other regenerated cellulose types, and lyocell excels in improved mechanical and chemical properties, while modal exhibits enhanced strength and durability properties if compared to viscose [43].

The regenerated cellulose fibers are highly hydrophilic because of the hydroxyl groups (–OH) on the cellulose chain. This property enhances their ability to absorb and retain moisture and makes them suitable for medical applications like wound dressing and surgical sutures where moisture management is a key parameter [122,123].

Regenerated cellulose fibers exhibit moderate chemical stability. They can be affected by strong acids and alkalis, but they generally maintain their integrity in physiological conditions, which is advantageous for medical use [123]. Regenerated cellulose fibers are insoluble in water, but can be dissolved in certain solvents, e.g., lyocell dissolves in cuprammonium hydroxide and N-Methylmorpholine N-oxide (NMMO), and viscose dissolves in sodium hydroxide. The solubility characteristic is exploited in the fiber production process [15,124,125]. But these fibers exhibit limited solubility in biological fluids, which makes them suitable for medical applications, like wound dressing [126].

The fibers of regenerated cellulose decompose naturally under environment conditions due to the presence of cellulose. This property is particularly beneficial for medical applications as post-use disposal is a consideration [126]. The biocompatibility of regenerated cellulose fibers is a key property of medical applications [127].

Regenerated cellulose fibers decompose at temperatures typically ranging from 200 °C to 300 °C. The exact decomposition temperature can vary depending on the fiber type and degree of polymerization, e.g., lyocell fibers usually have a higher thermal stability compared to viscose [128]. The glass transition temperature (Tg) of regenerated cellulose fibers is influenced by their molecular weight and crystallinity. These fibers generally exhibit a Tg around 250 °C [129,130].

Regenerated cellulose fibers each have distinct properties, which make them versatile for various textile applications, from everyday clothing to high-end fashion. Influenced by their production processes and chemical treatments, the following properties can be observed [32,120,131]:

- Viscose rayon: Softness, good dyeability, and moderate strength.
- Modal: Stronger, more durable, and excellent moisture management.
- Lyocell (Tencel): High strength, environmentally friendly, and excellent moisture control.
- Acetate: Luxurious feel, moderate moisture absorption, and lower strength.
- Cuprammonium (Cupro): Silk-like texture, good drape, and moderate strength.

### 3.1. Viscose Rayon Fibers

Viscose rayon, a type of regenerated cellulose fiber, exhibits lower crystallinity compared to natural cellulose fibers like cotton. The crystallinity of viscose rayon depends on the specific manufacturing conditions and post-treatment processes. The process disrupts the highly ordered crystalline structure of the original cellulose, leading to a more amorphous arrangement in the resulting fibers. However, the lower crystallinity also means that viscose rayon fibers are generally less strong and durable compared to natural cellulose fibers like cotton, especially when wet, and they may exhibit lower resistance to mechanical and environmental stresses [77,132].

The properties imparted by this level of crystallinity in viscose rayon include the following [58,132]:

- Softness and drapability: The lower crystallinity contributes to a softer and more drapable fabric, making viscose rayon ideal for clothing and textiles that require a fluid drape.
- Enhanced dyeability: The increased amorphous regions in viscose rayon fibers allow for better dye penetration, resulting in vibrant and uniform colors.
- Moisture absorption: Viscose rayon has good moisture-wicking properties, similar to cotton, providing comfort in clothing.
- Versatility: Its blend of properties makes viscose rayon suitable for a wide range of applications, from fashion garments to home textiles.

### 3.2. Modal Fibers

Modal is a type of regenerated cellulose fiber similar to viscose rayon but produced through a modified process that results in higher crystallinity and improved properties.

The increased crystallinity of modal fibers imparts several advantageous properties [133,134]:

- Strength and durability: Higher crystallinity leads to increased tensile strength and durability, making modal fibers more robust and long-lasting. Modal fibers are stronger and more durable than viscose rayon, both wet and dry.
- Softness and smoothness: Despite the higher crystallinity, modal fibers retain a smooth, silky texture and soft hand feel, which is ideal for high-quality textiles and apparel, often used in premium fabrics.
- Dimensional stability: Modal fibers exhibit better dimensional stability, maintaining their shape and size after repeated washing and drying.
- Moisture absorption: Modal fibers have excellent moisture-wicking properties, providing superior comfort and breathability in clothing. Modal has excellent moisture-wicking properties, superior to cotton and viscose.
- Color retention: The enhanced structure allows modal fibers to hold dyes well, resulting in vibrant, long-lasting colors.

These properties make modal fibers a popular choice for a variety of textile applications, including underwear, activewear, and bed linens, where both comfort and durability are desired.

### 3.3. Lyocell (Tencel^®^) Fibers

Lyocell has similar strength as polyester and it is stronger than cotton or all other man-made staple cellulosic fibers. It also has a very high dry and wet modulus in both the dry and wet states. All man-made cellulosic fibers lose strength and modulus when wetted, but lyocell reduces by much less than the others. However, the fibers do fibrillate during wet abrasion and thus, specific finishing techniques are required to achieve the best results.

Tencel^®^ can be processed via the established yarn manufacturing routes, using conventional machinery with few major changes to settings or procedures. Tencel^®^ possesses a non-durable crimp; it has a high modulus and there is little fiber entanglement. Thus, Tencel^®^ will open easily with little nep and yield yarns with high tensile strength and few imperfections. Tencel^®^ blends well with other fibers, especially other cellulosic fibers. It adds strength to the final yarn and enhances the performance and aesthetic values of the final fabrics [54].

Lyocell is a type of regenerated cellulose fiber known for its high crystallinity and eco-friendly production process. The higher crystallinity is attributed to the solvent-spinning process used in its production, which involves dissolving cellulose in a non-toxic solvent (N-methylmorpholine N-oxide or NMMO) and then regenerating the cellulose fibers in a coagulation bath. The higher crystallinity of lyocell/Tencel^®^ fibers results in several beneficial properties [93,135,136]:

- Strength and durability: The high degree of crystallinity provides lyocell fibers with exceptional tensile strength and durability, making them more resilient to wear and tear. The value of lyocell fiber tenacity is larger than for viscose and modal fibers, and is almost equal to polyester fiber. Lyocell is the only regenerated cellulose fiber with a wet tensile strength reaching the cotton wet strength. Lyocell has a significantly reduced elongation compared to viscose, but slightly above modal fibers.
- Softness and comfort: Despite their strength, lyocell fibers are also known for their smooth, soft texture, which enhances comfort in textiles and apparel. Lyocell fibers feature a fibrillar structure with microfibrils aligned parallel to the fiber axis because of a high degree of cellulose crystallinity, which allows lyocell fiber to easily develop a fibrillated surface under mechanical abrasion. Due to the high cellulose crystallinity produced via lyocell spinning, the moisture regain of lyocell fiber is slightly lower than for viscose.
- Moisture management: Lyocell fibers exhibit excellent moisture-wicking abilities, which help in maintaining dryness and comfort, making them suitable for activewear and intimate apparel.
- Biodegradability: The natural cellulose base and environmentally friendly production process contribute to the biodegradability of lyocell fibers, making them a sustainable choice.
- Sustainability: The solvent used in the production process is non-toxic and is recycled in a closed-loop system, making Tencel a more sustainable and environmentally friendly fiber.
- Versatility: The combination of strength, softness, and moisture management makes lyocell suitable for a wide range of applications, from fashion to home textiles.
- Environmental impact: The production process is more sustainable using a closed-loop system that recycles solvents.

These properties, driven by the high crystallinity of lyocell, contribute to its popularity as a premium, sustainable textile material.

### 3.4. Cuprammonium (Cupro) Fibers

Cuprammonium fibers, commonly known as cupro, are a type of regenerated cellulose fiber produced using the cuprammonium process. Regarding their crystallinity, cupro fibers generally exhibit lower crystallinity compared to natural cellulose fibers but higher crystallinity than some other types of regenerated cellulose fibers, such as viscose rayon. This intermediate level of crystallinity imparts cupro fibers with several desirable properties, including a silky texture, good drape, and a soft hand feel. Additionally, the lower crystallinity compared to natural fibers results in improved dyeability and a smoother surface, which enhances their suitability for high-quality textiles and apparel applications [58,137]:

- Softness and smoothness: Cupro fibers are known for their silk-like feel and smooth texture.
- Moisture absorption: Good moisture-wicking properties.
- Drape: Excellent drape and fluidity.
- Strength: Generally weaker compared to lyocell, but stronger than some types of viscose.

### 3.5. Cellulose Acetate Fiber

Acetate fibers, also known as cellulose acetate, typically exhibit lower crystallinity compared to other cellulose fibers like cotton, modal, or lyocell. It is no longer considered a regenerated cellulose fiber because the polymer formula to form acetate fiber is acetate (cellulose ester) instead of cellulose. The relatively low crystallinity is due to the chemical modification of cellulose during the production process. In this process, cellulose is acetylated by reacting it with acetic anhydride, resulting in cellulose acetate. This modification reduces the ability of the cellulose chains to align and form crystalline regions, leading to a more amorphous structure.

The properties imparted by this level of crystallinity in acetate fibers include the following [58]:

- Softness and drapability: The low crystallinity contributes a soft, smooth texture and excellent drapability, making acetate suitable for lightweight, flowy fabrics. Acetate fibers have a luxurious feel and a high sheen, and excellent drapability and fluidity.
- Sheen and luster: Acetate fibers have a natural sheen and luster, giving fabrics made from acetate an attractive, silky appearance.
- Moisture absorption: While not as absorbent as more crystalline cellulose fibers, acetate still has moderate moisture absorption, providing some level of comfort. It is moderate, though less effective compared to cotton and lyocell.
- Color retention: Acetate fibers take dyes well and retain color vibrantly, which is beneficial for fashion and decorative textiles.
- Resistance to shrinkage and wrinkling: The chemical structure of acetate fibers provides good resistance to shrinkage and wrinkling, enhancing their durability and ease of care.
- Strength: Lower tensile strength compared to other regenerated cellulose fibers.

However, the lower crystallinity also means that acetate fibers are generally less strong and durable compared to higher crystallinity cellulose fibers. They may also have lower resistance to heat and chemical damage.

## 4. Medical Applications of Main Regenerated Cellulose Fibers

Medical textiles are one of the fastest-growing sectors of technical textiles. The product range in this sector encompasses a wide array of items, spanning from disposable healthcare and hygiene products (e.g., napkins and diapers) to specialized products including operating room textiles, sutures, scaffolds, and sensors. The selection of fibers and fabrics for these applications varies significantly based on the specific property requirements of the medical textile raw materials. Medical textile products demand biocompatibility (biostability or biodegradability), non-toxicity and nonallergenic, absorbency and softness, elasticity and flexibility, and relative mechanical properties (tenacity, strength, durability, and sterilizability) [138].

Medical textile products are classified into four categories: (1) non-implantable materials, (2) implantable materials, (3) extracorporeal devices, and (4) healthcare and hygiene products [139,140,141,142].

Non-implantable materials are designed exclusively for external application in the human body. Their primary functions include protecting the skin from external infections, absorbing body fluids, or delivering medication to damaged skin. Non-implantable materials encompass wound dressings, plasters, bandages, gauze, and compression bandages, among others. These materials are essential for wound healing, providing protection against infection, and absorbing blood and exudates. These products are typically manufactured from various types of textile fibers, selected based on specific application requirements and end uses. [142,143,144]

The inherent comfort of cellulose-based materials like cotton and regenerated cellulosic fibers (viscose rayon) makes them ideal for non-implantable textile products (see Table 3). The porous structure of cellulose-based fibers provides excellent moisture-wicking capabilities, and it also has unique characteristics such as biodegradability and ease of dyeing, in addition to its being soft and comfortable [145,146]. Regenerated cellulose fibers are widely used in non-implantable materials due to their versatile properties, such as softness, absorbency, and environmental sustainability. These fibers are applied in various non-medical and industrial contexts.

Regenerated cellulose fibers are versatile and used in a wide range of non-implantable materials:

- Viscose rayon: Common in textiles and nonwovens for its softness and absorbency. Viscose rayon fibers are used as wound dressings due to their absorbency and comfort and are employed in surgical drapes and gowns only in non-critical settings for their softness and cost-effectiveness.
- Lyocell (Tencel): Used in apparel and home textiles for its strength and moisture management. Lyocell fibers are advanced wound dressings due to biocompatibility and moisture management. As materials for surgical gowns and drapes, lyocell fibers are used in high-performance medical textiles.
- Modal: Employed in underwear and towels for its softness and absorbency. Modal fibers are used in high-performance medical textiles such as patients’ gowns and beddings, and for wound dressings due to softness and moisture management.
- Acetate: Used in fashion fabrics and linings for its luster and drape. Used in some medical linens for its softness.
- Cuprammonium fibers (cupro): Applied in luxury textiles and some technical fabrics for their softness and breathability. Used in high-end medical linens due to their softness—for luxury medical linens. Emerging applications due to their sustainability and performance for medical textiles.

Implantable material textiles are materials designed for use within the human body or beneath the skin. The primary requirement for these applications is biocompatibility, versatility, and suitability for medical applications. These materials are generally used for repairing damaged internal organs, skin, or wounds, particularly during surgery. They can be categorized into soft tissue implants, orthopedic implants, and cardiovascular implants. Soft tissue implants are used in areas such as ligaments and cartilage. Orthopedic implants function as artificial bones, and cardiovascular implants are employed in heart valves and vascular grafts (see Table 4). Surgical sutures, which are commonly used to close skin wounds, also fall under this category. The fibers used in these applications range from natural materials like silk and biodegradable polymers such as chitin, collagen, and chitosan, to synthetic polymers including polyester, polyamide, polyethylene, and polytetrafluoroethylene [143,144,147].

Regenerated cellulose fibers are employed in various implantable materials due to their properties:

- Viscose rayon: Used in sutures and surgical meshes for its absorbability and biocompatibility, but in general, the use of viscose is limited and usually not used for implantable materials due to lower durability and potential for degradation.
- Lyocell (Tencel): Ideal for tissue engineering scaffolds and surgical meshes due to its strength and biocompatibility. Lyocell fibers are utilized in scaffolds for tissue regeneration.
- Modal: Applied in implantable textiles and surgical dressings for its softness and flexibility. Generally not used for implantable materials or is limited in use.
- Acetate: Utilized in controlled release systems and biodegradable meshes for its biodegradability and film-forming capabilities. Limited used, not typically used for implantable materials due to lower durability.
- Cuprammonium fibers (cupro): Used in tissue engineering and surgical textiles for its softness and smoothness. Rarely used for implantable materials—limited use.
- Ioncell: Investigated for advanced applications in tissue engineering and surgical meshes due to its high strength and biocompatibility. Potential for implantable materials because of biocompatibility.

These fibers provide various benefits in implantable materials, enhancing performance and patient outcomes.

Analogous to implantable textiles, extracorporeal devices are implantable artificial organs composed of textile materials. These artificial organs are utilized within the human body to support vital organ functions, including artificial kidneys, livers, and lungs. These devices are used to replace or support the function of diseased organs. The primary function of these artificial organs is to purify the blood during bodily functions through processes such as dialysis, filtration, and adsorption [93]. Mostly synthetic fibers are used for such products, but regenerated fibers are employed as well (see Table 5).

Regenerated cellulose fibers have been employed in extracorporeal devices due to their favorable properties, such as biocompatibility, high absorbency, and structural integrity. Extracorporeal devices are medical devices that perform functions outside the body, such as in dialysis machines and blood filtration systems.

Regenerated cellulose fibers are crucial in extracorporeal devices due to their properties:

- Viscose rayon: Used in dialysis membranes and blood filtration due to high absorbency and biocompatibility, but general viscose fibers are not commonly used in extracorporeal devices.
- Lyocell (Tencel): Preferred for its strength and excellent biocompatibility in dialysis and blood filtration. The protectional applications of lyocell fibers for emerging uses in extracorporeal devices due to biocompatibility.
- Modal: Applied in specific blood filtration systems with good moisture absorption, but in general, not commonly used in extracorporeal devices or in limited use.
- Acetate: Utilized in niche applications for its softness and moderate moisture management. Not commonly used or in limited application.
- Cuprammonium fibers (cupro): Employed in high-performance dialysis membranes and specialized blood filtration systems due to their smooth texture. Limited application in extracorporeal devices, not commonly used.
- Ioncell: Emerging uses—potential applications in extracorporeal devices.

These fibers offer different advantages depending on the specific requirements of the extracorporeal devices, enhancing performance and patient safety.

Healthcare and hygiene products are designed to protect both medical personnel and patients from infections. They can be either washable or disposable after use. This category includes items ranging from personal protective clothing to disposable masks. Additionally, personal hygiene products such as napkins, tissues, hospital bed linens, uniforms, and diapers fall under this category. Using regenerated cellulose fibers in healthcare and hygiene products includes face masks, personal protective equipment (PPE), and sanitary products. The COVID-19 pandemic significantly increased the demand for these products. The primary requirements for these textiles are that they must be nonallergenic, non-toxic, and non-carcinogenic. Nonwoven materials, often made from regenerated cellulose fibers, have been widely used due to their breathability, strength, and bacterial resistance [144,148,150,151].

Cellulose-based materials like cotton and regenerated cellulosic fibers (viscose rayon) are predominantly used in medical applications due to their high moisture absorbency and comfort properties, which makes them ideal for hygienic and healthcare clothing (see Table 6). The porous structure of cellulose-based fibers provides excellent moisture-wicking capabilities, along with a soft handle and drape, which identifies it as a skin-friendly material [145,146].

Regenerated cellulose fibers are extensively used in healthcare and hygiene products due to their excellent absorbency, softness, and biocompatibility. These fibers are commonly found in items such as wound dressings, surgical drapes, hygiene products, and more.

A summary of the medical application of conventional regenerated cellulose fibers is presented in Table 7.

Regenerated cellulose fibers offer numerous benefits for healthcare and hygiene products:

- Viscose rayon: Used in wound dressings, surgical drapes, and hygiene products for its absorbency and softness, and because of its comfort and absorbency, viscose fibers are utilized in hygiene products, such as sanitary pads and medical linens.
- Lyocell (Tencel): Applied in advanced wound care and hygiene products for its moisture management and biocompatibility. These fibers are used for hospital bedding and garments because of their comfort and performance.
- Modal: Used in hygiene products and medical textiles for its softness and absorbency; for sanitary pads and medical linens, these fibers are used for their high absorbency and comfort.
- Acetate: Utilized in wound dressings and some hygiene products for its softness and moisture management. Occasionally used in nonwoven hygiene products (sanitary products).
- Cuprammonium fibers (cupro): Employed in healthcare textiles and premium hygiene products for their softness and biocompatibility. Employed in certain high-end healthcare textiles.

These fibers provide various advantages in healthcare and hygiene applications, enhancing the performance and comfort of these products.

## 5. The Newest Regenerated Cellulose Fibers Used for Medical Applications

The newest regenerated cellulose fibers used for medical applications are typically advanced variations in traditional cellulose fibers, incorporating innovations in production techniques and functional properties to meet specific medical needs. Some latest developed regenerated cellulose fibers used for medical applications are as follows:

- Nanocellulose fibers: Fibers are produced by breaking down cellulose into nanoscale dimensions. This can be performed through chemical, mechanical, or enzymatic methods. These fibers exhibit extraordinary mechanical properties, high surface area, and biocompatibility. These properties contribute to their suitability for various biomedical applications, such as wound dressings, tissue engineering scaffolds, and drug delivery systems. Applications: used in wound dressings, drug delivery systems, and tissue engineering scaffolds due to their high strength and ability to support cell growth [155,156].
- Regenerated cellulose nanofibers (RCNFs): RCNFs are produced using advanced methods to extract and refine cellulose fibers to the nanometer scale. They offer enhanced mechanical properties, high surface area, and improved interactions with biological tissues. These properties enhance their suitability for various medical applications, including wound care, tissue engineering, and drug delivery systems. Application: utilized in advanced wound care products, tissue engineering, and as carriers for drug delivery systems [157,158].
- Bioactive cellulose fibers: Bioactive cellulose fibers are engineered to incorporate active agents such as antimicrobial or anti-inflammatory agents within the cellulose matrix. These fibers provide additional therapeutic benefits beyond the structural support of traditional cellulose. Bioactive cellulose fibers are designed to have specific properties that enhance their performance in medical applications. These fibers are often functionalized with bioactive agents to provide additional therapeutic benefits, such as antimicrobial properties or enhanced healing. These properties contribute to their effectiveness in various medical applications, such as wound care, drug delivery systems, and implants. Applications: employed in wound dressings, surgical sutures, and implants to enhance healing and reduce infection [159,160].
- Electrospun cellulose nanofibers: Electrospinning techniques are used to produce ultra-fine cellulose fibers with diameters in the nanometer range. These fibers have high surface area–volume ratios, which are beneficial for medical applications. Electrospun cellulose nanofibers, produced using electrospinning techniques, possess specific properties that make them highly suitable for medical applications. These properties are crucial for their performance in areas such as tissue engineering, wound healing, and drug delivery. These properties make electrospun cellulose nanofibers highly effective for applications in tissue engineering, wound care, and drug delivery systems. Applications: used in creating scaffolds for tissue engineering, drug delivery systems, and wound care products [161,162].
- Lyocell-like fibers with enhanced functionalization: Recent advancements in lyocell-like fibers involve functionalizing the fibers with additional properties such as enhanced biocompatibility, controlled drug release, or specific mechanical attributes tailored for medical use. Applications: employed in a range of medical textiles, including wound dressings, surgical gowns, and drug delivery systems [163].
- Ioncell is a relatively new type of regenerated cellulose fiber produced using an ionic liquid process. The circularity of Ioncell fibers is generally high, reflecting their more regular cross-sectional shape compared to other regenerated cellulose fibers. Ioncell is a type of regenerated cellulose fiber produced using an ionic liquid-based process. This innovative process offers several advantages over traditional methods. The crystallinity of Ioncell fibers is typically high, often ranging from 50% to 60%. This high crystallinity is achieved through the controlled dissolution and regeneration process, which promotes the formation of well-ordered crystalline regions within the fiber. Applications: Applied in advanced hygiene products and medical textiles for their strength and durability. Used in certain medical textiles (healthcare textiles) for their properties [164,165].

The main properties of some new regenerated cellulose fibers, described above, are presented in Table 8.

The newest regenerated cellulose fibers for medical applications represent significant advancements in fiber technology, focusing on enhancing properties such as strength, biocompatibility, and functionality.

## 6. Recycling and Challenges of Regenerated Cellulose Fibers for Medical Applications

The industry of regenerated cellulose fibers, including materials like viscose and lyocell, has shown significant growth in various medical applications from 2020 to 2024. The medical textile market, including the segments mentioned above, was valued at approximately USD 24.7 billion in 2020 and is expected to grow at a compound annual growth rate (CAGR) of around 3% from 2021 to 2028. This growth is attributed to the rising demand for medical textiles in various applications, increasing healthcare awareness, and advancements in textile technology [144,150,151]

Overall, the industry for regenerated cellulose fibers in medical applications has seen robust growth driven by technological advancements, increasing healthcare needs, and a heightened focus on hygiene and safety, particularly due to the COVID-19 pandemic.

In general, all regenerated cellulosic fibers offer many advantages for medical applications, including biocompatibility, absorbency, and comfort. However, their use in medical settings comes with several challenges (see Table 9).

The recycling of regenerated cellulose fibers used in medical applications is a significant area of research due to the increasing focus on sustainability and reducing environmental impact [71,166,167,168]. Regenerated cellulose fibers are valuable in medical textiles, but their disposal and recycling present challenges. RCF-based products have the potential to be recycled into new fibers or repurposed into other products, contributing to a circular economy in healthcare. This can help minimize the need for virgin resources and reduce overall waste.

Regulations on recycling regenerated cellulose fibers for medical purposes emphasize safety, environmental care, and the overall impact. These laws ensure that recycled fibers comply with rigorous health and safety standards for medical use, reducing risks to patients and medical staff. In most jurisdictions, regenerated cellulose fibers intended for direct or indirect use in or on the human body (e.g., wound dressings and surgical sutures) are classified as medical devices. This classification places them under stringent regulatory oversight. The classification levels are as follows:

- Class I (low risk): Basic dressings made from regenerated cellulose may fall under this classification.
- Class II (moderate risk): Absorbable regenerated cellulose sutures and hemostatic agents may fall under this classification.
- Class III (high risk): Regenerated cellulose materials used in critical applications, like internal implants, may require this classification.

Regulations also support sustainable recycling practices that cut waste and save resources while limiting environmental damage from the chemicals or byproducts of fiber processing. Ultimately, these regulations strive to balance advances in fiber recycling with strict standards for health, safety, and environmental responsibility. When used in medical products (like wound dressings or sutures), regenerated cellulose fibers must meet health standards set by authorities, such as the FDA in the U.S. and the EMA in Europe. These fibers also need to comply with strict biocompatibility and safety standards (e.g., ISO 10993) to ensure safe use in or on the human body. Manufacturers must implement quality control systems according to standards like ISO 13485 to meet medical device production standards [171].

For recycling and waste management, the EU’s Waste Framework Directive mandates recycling and waste management practices for textiles, including cellulose fibers, to reduce landfill waste. In some regions, Extended Producer Responsibility laws require companies to manage their products’ post-consumer waste, encouraging recycling and reuse programs for cellulose-based textiles. Regulations like the EU’s REACH program restrict hazardous chemicals in fiber production to protect the environment and ensure chemical safety [172].

Countries like those in the EU promote sustainable textile policies, like a circular economy, including cellulose fibers, by supporting recycling and reuse, encouraging fiber innovation, and investing in recycling infrastructure. Regions such as the EU have established textile recycling targets and mandatory collection systems (beginning in 2025) to ensure cellulose-based textiles are recycled instead of landfilled [172].

These regulations guide the safe and sustainable production, use, and disposal of regenerated cellulose fibers, supporting both medical and environmental goals.

### 6.1. Recycling of Viscose Rayon Fiber

Viscose rayon can be recycled via chemical or mechanical processes. The chemical recycling process involves dissolving viscose rayon in a solvent and then reconstituting the cellulose into new fibers or products. This process is complex because it involves breaking down the fibers into their chemical components and then reprocessing them again into fibers. This method can be more effective in maintaining fiber quality but is costly. The mechanical recycling of viscose involves shredding the fibers and re-spinning them into new products, though this is less common for medical-grade viscose due to contamination issues. However, this process often results in quality degradation (loss of such properties as strength, flexibility, and comfort), which can be problematic for applications requiring high performance, such as medical textiles [89,173,174,175].

A specific challenge of viscose rayon recycling is that medical-grade viscose often comes into contact with bodily fluids and contaminants, complicating recycling processes as fibers need to meet strict hygiene and sterilization requirements. The effective sterilization of viscose rayon fibers is a significant challenge.

Innovations in the recycling technologies of viscose rayon fibers, such as enzyme-based recycling methods, might potentially improve the efficiency and effectiveness of recycling viscose fibers. Developing closed-loop recycling systems where viscose fibers are continuously recycled within the same supply chain could enhance sustainability and reduce waste [173,174].

### 6.2. Recycling of Lyocell (Tencel^®^) Fibers

Lyocell fibers are well suited to closed-loop recycling systems where the fibers are dissolved and re-spun into new lyocell fibers. This process is relatively efficient and retains the fiber’s original properties. Similarly to closed-loop systems, lyocell can be chemically recycled by dissolving and regenerating cellulose. Lyocell fibers can be mechanically recycled as well; that is, fibers are ground into smaller pieces and then re-spined. But, the recycling of lyocell fibers faces some challenges. The closed-loop process can be more expensive compared to chemical and mechanical recycling methods. Ensuring that chemically recycled lyocell maintains the same quality as virgin fibers is a key challenge. However, this method is more complex and expensive compared to mechanical recycling, but the mechanical recycling method can cause degradation in fiber quality, which may be problematic for medical applications where fiber integrity is crucial [15,50,91,175].

Specific challenges for medical applications for recycled lyocell and Tencel^®^ fibers are that they must meet stringent hygiene and sterilization standards for medical textiles and the recycling process needs to ensure that fibers are free from contaminants and can be properly sterilized.

As for lyocell fibers, an innovation in the recycling process, such as enzyme-based recycling and more efficient chemical processes, could improve the effectiveness and efficiency of recycling lyocell and Tencel^®^ fibers. Implementing closed-loop recycling systems, where fibers are continuously recycled within the same supply chain, could enhance sustainability and reduce waste [175,176].

### 6.3. Recycling of Modal Fibers

Modal fibers can be recycled chemically or mechanically. Chemical recycling is similar to lyocell, where they are dissolved and reconstituted. While this method can preserve the quality of fibers better than mechanical recycling, it is complex and often more expensive. Modal fibers can be mechanically recycled into nonwoven fabrics or other products, but contamination from medical use can limit this method. As with other medical regenerated cellulose fibers, the presence of contaminants can complicate the recycling process. But the primary challenge is that mechanical recycling (shredding the fibers and re-spinning them into new yarns) can degrade the fiber quality, which is critical for high-performance applications such as medical textiles. Recycled modal fibers need to maintain their performance characteristics, such as strength, elasticity, and moisture management, which are important for medical textiles that must perform reliably in various conditions [71,175,177].

Advanced recycling technologies, such as enzyme-based recycling methods or closed-loop systems, can enhance the efficiency and effectiveness of recycling modal fibers, potentially addressing some challenges associated with traditional methods. Developing circular economy models for modal fibers, where fibers are continuously recycled within the supply chain, can improve sustainability and reduce waste [167].

### 6.4. Recycling of Cupro Fibers

As other regenerated cellulose fibers, described above, cupro fibers can be recycled in mechanical or chemical way. Mechanical recycling may lead to degradation in fiber quality due to shredding and re-spinning cupro fibers. The chemical recycling process can be complex and may have environmental implications due to the chemicals used. Specific challenges for the medical applications of recycled cupro fibers are the removal of contaminants and ensuring effective sterilization [71,168].

Advanced recycling technologies, such as solvent-based recycling and closed-loop systems, may improve the efficiency and sustainability of recycling cupro fibers. Developing circular economy models where cupro fibers are continuously recycled within the supply chain could enhance sustainability and reduce waste [178].

### 6.5. Recycling of Acetate Fibers

The mechanical recycling process of acetate fibers involves grinding the fibers and re-spinning them. This process is the same as for the regenerated cellulose fibers and can lead to a loss of fiber quality, which can be problematic in medical textiles. Also, it leads to challenges in removing contaminants, allowing the effective sterilization of the fibers, and ensuring that recycled acetate fibers maintain their initial properties, such as strength, flexibility, and moisture management [175,179,180].

As for cupro fibers, solvent-based recycling and improved chemical processes could enhance the efficiency and sustainability of recycling acetate fibers, and closed-loop systems where acetate fibers are continuously recycled within the supply chain could improve sustainability and reduce waste [179,180].

Regenerated cellulose fibers have become an exciting area of research and application in healthcare, driven by their biocompatibility, sustainability, and versatility. These fibers include varieties like lyocell, viscose, modal, cupro, and acetate, each bringing unique characteristics to medical textiles and biomaterials. Their potential to transform medical technologies is significant, particularly in areas such as tissue engineering, wound care, drug delivery, and sustainable healthcare solutions. A summary of the benefits and emerging technologies of regenerated cellulose fibers for medical applications is presented in Table 10.

## 7. Conclusions

Regenerated cellulose fibers are increasingly being explored for medical applications due to their desirable properties, such as biocompatibility, biodegradability, and comfort. The future of these fibers in medical textiles is shaped by several emerging trends and technological advancements. Future advancements are likely to focus on enhancing closed-loop production processes to make them more efficient and cost-effective. This includes better solvent recovery systems and reduced environmental impact. The integration of nanotechnology can be carried out to create nanofibers with enhanced properties, such as increased surface area, antimicrobial activity, and improved mechanical strength.

While RCFs are known to be biocompatible, there is a lack of long-term clinical data on how these fibers perform over extended periods, especially in complex medical applications such as implants or tissue scaffolds. Research should focus on the long-term effects of RCFs on tissue regeneration, their degradation rates, and their mechanical stability in demanding environments such as bone scaffolds or vascular implants. Developing reinforced RCF composites that can withstand higher mechanical stress while maintaining biodegradability could open up new applications in orthopedics and cardiovascular surgery. The functionalization of RCFs (e.g., with antimicrobial agents or drug-loaded fibers) has shown promise, but cost-effective manufacturing processes for large-scale production remain a challenge. The high cost of incorporating agents like silver nanoparticles can limit the use of these advanced materials in lower-resource settings. Creating affordable antimicrobial RCF-based dressings that can be deployed in global healthcare markets, especially in areas with limited access to expensive wound care technologies, presents a significant opportunity. Smart textiles using RCFs with embedded biosensors have shown great potential, but their scalability and commercial viability are still in question. The integration of electronics into cellulose-based textiles poses challenges related to durability, data transmission, and cost. There is a need for clearer regulatory frameworks and standards for the use of RCF-based medical products, especially in emerging applications like tissue engineering scaffolds and drug delivery systems. Navigating the regulatory approval process can be slow and complex, delaying the commercialization of innovative RCF products.

## Figures and Tables

**Table 1:** The advantages of regenerated cellulose fibers, used for medical applications, compared to synthetic and natural fibers.

Property	Regenerated Cellulose Fibers (Viscose, Lyocell, Modal, and Acetate)	Synthetic Fibers (Polypropylene and Polyester)	Natural Fibers (Cotton and Silk)	Scientific References
Cost-effectiveness	Moderate cost, relatively inexpensive production due to large-scale manufacturing.	Low cost, very cheap due to petrochemical origin and established production lines.	High cost for premium fibers like silk; cotton can vary in price depending on the quality.	[19,20,21]
Biodegradability	Highly biodegradable (especially lyocell and viscose); reduces environmental impact in disposable medical products.	Non-biodegradable; contributes to long-term environmental pollution (plastic waste).	Biodegradable, but cotton production has a significant environmental footprint (e.g., water usage).	[19,20,21]
Biocompatibility	Excellent biocompatibility due to cellulose origin; minimal risk of allergic reactions or irritation.	Variable; some synthetic fibers may cause skin irritation or inflammation, especially in sensitive patients.	High biocompatibility; natural fibers like silk are hypoallergenic, but cotton can sometimes be harsh on sensitive skin.	[21,22,23]
Absorbency	Excellent moisture absorption (e.g., lyocell and viscose); ideal for wound care and surgical dressings.	Poor moisture absorption; tends to repel liquids, which is useful in some protective textiles but not for wound care.	High absorbency (especially cotton); suitable for certain medical textiles but not as specialized as cellulose fibers.	[21,22,23]
Sterilization compatibility	Can be sterilized via autoclaving, gamma radiation, and other methods without losing structural integrity (except acetate).	Excellent for sterilization; resistant to degradation by most sterilization methods.	Natural fibers like cotton can be sterilized but may lose structural integrity or shrink over time. Silk is more delicate in sterilization.	[19,20,22]
Environmental sustainability	High sustainability (especially lyocell with closed-loop processing); lower chemical and energy inputs than synthetic fibers.	Poor sustainability due to petrochemical base and non-renewable resources used in production.	Mixed; cotton production is water-intensive, but silk is more environmentally friendly in small-scale production.	[19,20,23]
Patient comfort	Soft, breathable, and skin-friendly; suitable for long-term wear in medical garments and wound dressings.	Less breathable and can cause skin irritation; often uncomfortable for long-term skin contact (e.g., hospital gowns).	High comfort for silk, but cotton can sometimes cause friction on sensitive or healing skin.	[21,22,23]
Applications in the medical field	Used in wound dressings, surgical swabs, hygiene products, and biodegradable implants due to biocompatibility and absorbency.	Used in protective clothing, surgical masks, and other disposable medical products, but less suitable for direct skin contact.	Used in traditional bandages, sutures (silk), and some medical textiles, but less common in advanced medical applications.	[20,21,23]
Durability	Moderate durability; can be engineered for strength in specific medical applications (e.g., lyocell scaffolds).	High durability; long-lasting and tear-resistant, ideal for protective gear like surgical drapes.	Variable; silk is strong but delicate, while cotton is less durable in clinical use due to wear and tear.	[19,20,22]
Antimicrobial functionalization	Can be functionalized with antimicrobial agents (e.g., silver and iodine) for advanced wound care applications.	Often treated with antimicrobial agents, but additives may leach over time or be toxic.	Silk has natural antibacterial properties, while cotton may require treatment to gain antimicrobial properties.	[19,21,22]

**Table 2:** Properties of cotton and some regenerated cellulose fibers [50,70,77,120,121,122].

Characteristic	Cotton	Viscose Rayon	Modal	Lyocell	Tencel	Cupro	Acetate
Density, g/cm^3^	1.52	1.46–1.54	1.53	1.5	1.5	1.5	1.29–1.33
Tenacity, cN/tex	19.5–35	8.8–24	34	30–45	36–44	9–28	12.8
Moisture regain, %	7–8	11–14	11.8	10–13	11	11–12.5	6–7
Elongation, %	7–14	17–30	12	12–18	16–18	6–25	24
Crystallinity, %	60–70	30–40	40–48	50–65	50–65	30–40	20–30
Circularity of fiber cross-section *	0.4–0.8	0.5–0.8	closer to 1	approaching 1	approaching 1	0.7–0.9	Lower circularity

Note: *—where 1 represents a perfect circle.

**Table 3:** Regenerated cellulose fibers used for non-implantable materials [147,148].

Type of Regenerated Cellulose Fibers Used	Application Areas
Viscose and lyocell	Absorbent pad for wound care
Viscose, lyocell, and modal	Wound-contact layer
Viscose	Base material for wound care
Viscose and lyocell	Base material for pads and bandages
Viscose and lyocell	Simple bandages
Viscose and lyocell	High-support bandages
Viscose and lyocell	Compression bandages
Viscose and lyocell	Orthopedical bandages
Viscose	Plasters
Viscose and lyocell	Gauze dressing
Viscose and cotton linters	Wadding
Cotton linters	Virus removal filter

**Table 4:** Regenerated cellulose fibers used for implantable materials [53,143,144,147].

Type of Regenerated Cellulose Fibers Used	Application Areas
Viscose	sutures
Viscose, lyocell, and Ioncell	surgical meshes, e.g., for hernia
Lyocell, cupro, and Ioncell	scaffolds for tissue engineering
Modal	surgical dressings
Acetate	controlled drug release systems
Acetate	biodegradable meshes
Cupro	surgical textiles

**Table 5:** Regenerated cellulose fibers used for extracorporeal devices [54,144,147,149].

Type of Regenerated Cellulose Fibers Used	Application Areas	Function
Hollow viscose	artificial kidney	remove waste products from patients’ blood
Hollow viscose	artificial liver	separate and dispose of patients’ plasma, and supply fresh plasma
Viscose, lyocell, and modal	hemodialysis membranes	the selective filtration of waste products from the blood
Viscose, lyocell, modal, and acetate	peritoneal dialysis	to facilitate the exchange of waste products and electrolytes through the peritoneal membrane
Viscose, lyocell, and modal	plasma filters	for removing proteins, toxins, and other unwanted substances from the blood
Viscose, lyocell, and modal	dialyzer units	for both hemodialysis and hemofiltration

**Table 6:** Regenerated cellulose fibers used for healthcare/hygiene products [54,144,147].

Type of Regenerated Cellulose Fibers Used	Application Areas	Structure of the Fabric
Viscose	Surgical caps	Nonwoven
Viscose	Surgical masks	Nonwoven
Superabsorbent fibers and wood fluff, modal, lyocell, and acetate	Absorbent layer for incontinence diaper/sheet	Nonwoven
Viscose and lyocell, modal, and cupro	Surgical swabs, drapes, and cloths/wipes,	Nonwoven

**Table 7:** Medical application of conventional regenerated cellulose fibers.

Fiber Type	Primary Medical Application	Advantages	Challenges	References
Viscose rayon	Wound care and surgical sponges	High absorbency and cost-effective	Residual chemicals from production	[21]
Cupro	Bandages and medical textiles	Soft and hypoallergenic	Copper residue may require further purification	[152]
Modal	Patient garments and hospital bedding	Durable and moisture-wicking	Moderate cost	[20]
Lyocell	Advanced wound dressings and tissue scaffolds	Biodegradable and high absorbency	Limited production capacity	[153]
Acetate	Medical packaging and drug delivery systems	Good barrier properties and biocompatible	Low absorbency	[154]

**Table 8:** Properties of some new regenerated cellulose fibers [155,156,157,158,159,160,161,162,163,164,165].

Characteristic	Nanocellulose Fiber	Regenerated Cellulose Nanofibers (RCNFs)	Bioactive Cellulose Fibers	Electrospun Cellulose Nanofibers	Lyocell-like Fibers	Ioncell
Density, g/cm^3^	1.5–1.6	1.5–1.6	1.5–1.6	1.5–1.6	1.48–1.52	1.5
Tenacity, cN/tex	1 × 10^8^–2 × 10^8^	1 × 10^8^–2 × 10^8^	0.8 × 10^8^–1.5 × 10^8^	0.5 × 10^8^–1.5 × 10^8^	40–60	20.5–36.5
Moisture regain, %	10–15	10–15	8–15	8–15	10–15	10–12
Elongation, %	5–10	5–10	5–10	5–10	10–20	7–13
Crystallinity, %	70–90	70–90	60–90	70–90	40–55	50–60
Circularity of fiber cross-section *	Close to 1	Close to 1	Close to 1	Close to 1	0.8–1	0.7–0.9.

Note: *—where 1 represents a perfect circle.

**Table 9:** Challenges of usage of regenerated cellulose fibers for medical applications [71,84,166,167,168,169,170].

Challenge	Description
Biocompatibility	Allergic reactions: Despite being generally biocompatible, some individuals may experience allergic reactions or sensitivities to regenerated cellulose fibers.
	Infection risk: Medical-grade regenerated cellulose fibers must be carefully processed to minimize the risk of infections or inflammatory responses.
Contamination and sterilization	Sterilization: Regenerated cellulose fibers used in medical applications must withstand sterilization processes (such as autoclaving or gamma irradiation) without degrading. Some fibers may degrade or lose their properties during these processes.
	Contamination: Ensuring the fibers are free from contaminants and pathogens is crucial, especially for applications like wound dressings and surgical materials.
Mechanical properties	Strength and durability: Some regenerated cellulose fibers may lack the mechanical strength and durability required for certain medical applications, such as surgical sutures and implants.
	Wear and tear: Medical textiles made from regenerated cellulose can be prone to wear and tear, which can impact their performance and safety.
Environmental impact	Environmental sustainability: The production of regenerated cellulose fibers involves the use of chemicals and processes that can have significant environmental impacts. Addressing the sustainability of these processes is crucial.
	Recycling: Recycling regenerated cellulose fibers, particularly those used in medical applications, poses challenges due to contamination and the need for specialized recycling systems.
Cost and economic viability	Cost: Regenerated cellulose fibers can be more expensive than other materials, particularly when high purity and specific properties are required for medical use.
	Economic viability: Balancing cost with the need for high-performance medical textiles can be challenging, especially in low-resource settings.
Process and quality control	Consistency and quality control: Ensuring consistency in fiber quality and performance is critical for medical applications, where variations can impact safety and efficacy.
	Manufacturing process: The complexity of manufacturing processes for regenerated cellulose fibers can affect their quality and performance.

**Table 10:** Challenges of usage of regenerated cellulose fibers for medical applications.

Category	Potential/Benefit	Emerging Technologies	Example	References
Tissue engineering and regenerative medicine	RCFs like lyocell and bacterial nanocellulose (BNC) serve as scaffolds due to their porosity, strength, and biocompatibility.	Three-dimensional bioprinting enables customizable tissue scaffolds for skin regeneration and bone tissue.	Lyocell is explored for bone scaffolds, reducing recovery times in orthopedic surgeries.	[181]
Smart wound dressings and wearable biosensors	Smart textiles with biosensors can monitor wound environment (pH, temperature, and moisture) in real time.	Wearable devices using RCFs with embedded electronics reduce the need for frequent inspections.	Lyocell-based smart wound dressings transmit real-time data to healthcare providers, reducing hospital visits.	[182]
Drug delivery systems	RCFs (e.g., cupro and lyocell) allow for localized, controlled drug release, improving patient compliance.	Drug-eluting fibers release therapeutic agents over time, aiding in chronic wound care.	Cupro fibers in curcumin-loaded dressings for diabetic ulcers promoting healing and offering sustained drug release.	[183]
Biodegradability and waste reduction	RCFs are biodegradable, breaking down without leaving harmful residues, and reducing medical waste.	RCFs in disposable medical textiles (e.g., gowns and bandages) help reduce plastic waste.	Lyocell gowns and drapes degrade faster than synthetic alternatives.	[184]
Sustainable production processes	RCFs like lyocell use closed-loop processes where chemicals are recovered and recycled, reducing environmental impact.	Closed-loop production recycles over 99% of the solvent used, making lyocell eco-friendly.	Lyocell fiber production is a model for sustainable practices, aligning with global healthcare eco-initiatives.	[185]

## Data Availability

The original contributions presented in the study are included in the article, further inquiries can be directed to the corresponding author.
